# Determinants of parents' experiences with outpatient child and adolescent mental health services

**DOI:** 10.1186/1752-4458-5-22

**Published:** 2011-09-15

**Authors:** Olaf Holmboe, Hilde H Iversen, Ketil Hanssen-Bauer

**Affiliations:** 1 Norwegian Knowledge Centre for the Health Services, P.O. Box 7004 St Olavs plass, 0130 Oslo, Norway; 2 Centre for Child and Adolescent Mental Health, Eastern and Southern Norway, P.O. Box 4623 Nydalen, 0405 Oslo, Norway; 3 Department of Research and Development, Division of Mental Health Services, Akershus University Hospital, 1478 Lørenskog, Norway

**Keywords:** User experiences, Parent satisfaction, Child and adolescent mental health services, National survey

## Abstract

**Background:**

Few studies have investigated how demographic, clinical and organizational characteristics influence parents' experiences with child and adolescent mental health services (CAMHS). The objective of this study was to determine the effects of these characteristics on parents' experiences using data from a large national postal survey.

**Method:**

A questionnaire was mailed to 17,871 parents or other primary caregivers whose children were attending 1 of the 86 outpatient CAMHS in Norway in 2006. Multiple regression analysis was used to explore the associations between demographic, clinical and organizational characteristics, and three scales of parents' experiences.

**Results:**

The questionnaire was completed by 7906 parents (46%). Organizational characteristics such as involvement of the parents in treatment and accessibility to the clinic explained most of the variation in all three scales of parents' experiences. Although the effects of demographic and clinical characteristics of the children in some instances were statistically significant, they only accounted for a small amount of the total explained variance.

**Conclusion:**

Accessibility to the clinic and involvement of the parents in treatment are much stronger predictors of parental experiences with outpatient CAMHS than are demographic and clinical variables. Accessibility and involvement are at least partly influenced by the clinics themselves, and hence parental satisfaction may be enhanced by making the clinics more accessible and by involving the parents/caregivers in the treatment.

## Background

Patient satisfaction and experiences are increasingly used as indicators of quality in health care. Parents are often an integral part of the treatment within the child and adolescent mental health services (CAMHS), and their opinion may be crucial to the engagement and continuation of treatment [1]. Few studies have investigated the associations between background variables and parents' reported experiences with CAMHS [2,3]. It has been suggested that the demographic and clinical characteristics of the patients--in addition to organizational data--are needed when investigating parental satisfaction with CAMHS [4,5]. Most studies have identified one or more variables that are significantly associated with parental satisfaction with aspects of CAMHS, but we are not aware of any variables that are significantly related to parental satisfaction with CAMHS across all studies, or of studies that have provided information on the explanatory power of such variables beyond their statistical significance.

The findings regarding parent satisfaction with CAMHS in the published literature is often contradictory. For example, some studies have found no relationship between demographic variables and parental satisfaction [4,6], while others have identified some statistically significant relationships. One study showed that fathers tended to be less positive than mothers [7], and two other studies found that the parents of older children were less satisfied than those of younger children [8,9]. The parents' age has been shown to be positively associated with their satisfaction with CAMHS [7,10]. Family composition has rarely been examined in studies of parent satisfaction with CAMHS. Single and remarried mothers reported greater changes in their child's behaviour and improved coping with their child's problems than did married mothers in one study [11], while parents of children living at home reported statistically significant higher levels of satisfaction than those with children living away from home in another [12]. Ethnic background has been shown to be only weakly correlated with parental satisfaction. Several studies found no statistically significant association between satisfaction and ethnic background [4,7,12-14], while Heflinger et al. [10] found that parents of black children were more satisfied with one aspect of the treatment.

There is some evidence that health status can influence patients' satisfaction with health services in general [15]; however, it is unknown whether this relationship holds true regarding parents' experiences with CAMHS. The type or severity of the child's mental problems seems to have little effect on the parent satisfaction with CAMHS [4,6,10], but one study found that the satisfaction with the treatment outcome was less among parents of children referred with externalizing symptoms than for those with internalizing symptoms [8]. There is also some evidence that the severity of the child's symptoms and the stress felt by the parents while caregiving is negatively associated with their satisfaction with the services [2,4,14], and Godley et al. [2] found this to be the best predictor of parent satisfaction with CAMHS. But again, another study found no such relationship [16]. Some studies have found a statistically significant correlation between improvement in the child's mental problems and the parent satisfaction with the services [1,6]. Finally, the length of treatment was found to be positively associated with satisfaction in some studies [7,8] but not in others [4,12].

In addition, administrative and organizational aspects of the clinic may affect patients' satisfaction with CAMHS. One study of outpatient CAMHS found that a longer waiting time from referral to the start of examination or treatment was associated with a lower level of satisfaction [8]. Furthermore, one study found that a higher frequency of consultations at the clinic increased the satisfaction scores [4], while the findings of another study did not support this [6].

In summary, the results from published studies that have explored the relationship between demographic, clinical and organizational variables on the one hand and the parents' experiences with outpatient CAMHS on the other are not consistent. The aim of this study was to determine the influence and explanatory power of demographic, clinical and organizational variables on parents' experiences in Norway, as measured using a national postal survey.

## Materials and methods

Norwegian CAMHS are applied to those aged < 18 years, are a part of the publicly funded National Health Service and are available regardless of parental income. Private services of this kind are very few in Norway and their influence is negligible. In 2006, CAMHS in Norway treated a total of 47,280 children and adolescents (4.3% of the population aged 0-17 years), mostly (46,214) as outpatients. By the end of 2006, 3507 full-time equivalent employees worked in the Norwegian CAMHS, of whom 1773 (51%) worked at outpatient units [17]. At the time of the present study there were 86 outpatient CAMHS units in Norway.

The project was approved by The Regional Committee for Medical Research Ethics. Dispensation from patient confidentiality was given by The Directorate for Health and Social Affairs.

### Sample

The study sample consisted of parents or primary caregivers in 17,871 families. To be included in the study, the child had to be less than 16 years old with at least one appointment at an outpatient CAMHS during the final 4 months of 2006. The sample comprised a maximum of 400 patients per clinic; participants were chosen randomly if the clinic had more than 400 patients during the study period. They received a questionnaire by mail, and were asked to return it in a prestamped envelope. Two reminders were sent to non-respondents.

We compensated for non-responses by dividing the sample into seven response homogeneity groups [18]. These groups were based on diagnosis, length of treatment, ethnic background and group of inclusion, and used as a basis for weighting for non-responses. Telephone interviews were conducted with a sample of nearly 400 randomly selected non-respondents using a short version of the questionnaire.

### Questionnaire and register data

The questionnaire was developed following a review of the international literature, interviews with parents attending two outpatient CAMHS units, discussions within an expert panel and pilot testing of the questionnaire [19].

The clinics transferred data regarding the child's name, address, gender, age, date of referral, date of treatment start and end, reason for referral, diagnoses, and mother's and father's ethnic backgrounds. From these data we computed the waiting time and length of treatment (in units of days), and grouped ethnic background and diagnosis into appropriate categories. The diagnoses at CAMHS are registered in a multiaxial system of six axes based on the International Classification of Diseases (ICD)-10 [20,21]. Axis 4 refers to somatic diseases and was excluded. Axis 6 refers to the child's level of social functioning as measured by the Global Assessment of Psychosocial Disability scale, rated from 0 (superior/good social functioning) to 8 (profound and pervasive social disability), and was kept as a separate variable. The main diagnoses on the remaining axes were grouped into one variable.

### Statistical analyses

Statistical analyses were carried out using SPSS 15. Exploratory factor analyses identified three scales in the questionnaire, all of which had satisfactory psychometric properties [19]. The first scale, "Relationship with health personnel", comprised eight items, including care, understanding, respectfulness, cooperation and enough time. The second scale, "Information and participation", comprised four items addressing information about the child's condition and treatment alternatives, and the parents' influence on treatment. The third scale, "Outcome", comprised three items regarding changes in the child's condition and social functioning. The items included in each scale and their response categories are shown in table 1.

**Table 1:** The scales and their respective single items

Scale	Items
**Relationship with health personnel**^a^	Were the health personnel thoughtful and considerate towards you?
	Did the health personnel understand your concerns as a parent/guardian?
	Were the health personnel thoughtful and considerate towards your child?
	Were the health personnel polite and respectful towards you?
	Did the health personnel speak to you in a way that was understandable?
	Did the health personnel take your views seriously?
	Did you get enough time for contact and conversation with the health personnel?
	Did the health personnel cooperate well with you?

**Information and participation**^a^	Were you asked to give your views about the choice of treatment program?
	Did you have an influence in the choice of treatment program?
	Did you receive information on the different types of treatment available to your child?
	Did you receive information on your child's psychological condition?

**Outcome**^b^	Compared with before treatment started at the outpatient clinic, how is your child's well-being now?
	Compared with before treatment started at the clinic, how does your child function in your family now?
	Compared with before treatment started at the clinic, how does your child function outside of your family now (at school, at nursery, among friends and other social situations)?

Response categories for single items included in the scales:

^a ^Not at all, To a small extent, To a moderate extent, To a large extent, To a very large extent

^b ^Much worse, A little worse, Neither better nor worse, A little better, Much better

Regression analyses were conducted with each of the three scales as dependent variables, and demographic, clinical and organizational variables as independent variables. The coefficients for all of the correlations between the independent variables were *r *< 0.4. We first conducted linear regressions to assess the relationship between the scales and each of the independent variables separately. Independent variables with *p *< 0.1 in the bivariate regression analyses were included in multivariate models, where the independent variables were grouped and entered in four blocks. This enabled us to assess the explained variation attributable to each group of variables in addition to the statistical significance of the independent variables' relationship to the scales. The first block included the child's age and gender. The second block included clinical data about the child, such as the grouped diagnosis variable, the Global Assessment of Psychosocial Disability score on axis 6 and whether the child had previously been in contact with CAMHS. Variables on the parents' background were entered in the third block, and organizational variables related to the clinics' accessibility, involvement of the parents and the parents' knowledge of the services were entered in the forth block.

## Results

The questionnaire was returned by 7906 parents or primary caregivers (46% of those sent the questionnaire), and a further 226 out of 395 parents (57%) responded during the telephone follow-up study. An analysis of this material revealed that the level of satisfaction was the same for the telephone and mail respondents [19]. These results, together with weighting for response propensity, led to the conclusion that the low response rate had not induced serious bias, and hence that generalization to the entire population was justified [19]. The characteristics of the respondents and the children are presented in table 2.

**Table 2:** Characteristics of parents/primary caregivers and their children

	*n*		%	Mean (SD)	Median
**Child's age in years**	7906			11.3 (3.22)	12

**Parents' age in years**	7693			40.3 (6.62)	40

**Child's gender**	7903	Female	37		
		Male	63		

**Diagnosis axes 1, 2, 3 and 5**^**a**^	7906	Hyperkinetic or conduct disorders (F90, F91, F92)	30		
		Emotional disorders (F3, F4, F5, F93)	18		
		Developmental disorders (F7, F8)	8		
		Psychosocial problems only (Axis 5)	9		
		No mental or behavioural disorders or psychosocial problems	8		
		Other mental or behavioural disorders (F0, F1, F2, F6, F94-F99)	7		
		Not coded or invalid code used	21		

**Axis 6 (Global Assessment of Psychosocial Disability scale)**^**a**^	4945	0 Superior/good social functioning	2		
		1 Moderate social functioning	17		
		2 Slight social disability	27		
		3 Moderate social disability	34		
		4 Serious social disability	16		
		5 Serious and pervasive social disability	3		
		6 Unable to function in most areas	1		
		7 Gross and pervasive social disability	0		
		8 Profound and pervasive social disability	0		

**Previously treated by CAMHS**	7651	Never	74		
		Once	11		
		More than once	14		

**Parents' gender**	7639	Female	85		
		Male	15		

**Marital status**	7700	Married	54		
		Cohabitant	19		
		Neither married nor cohabitant	27		

**Household's highest education level**	7787	Primary school	7		
		High school	41		
		University graduate	30		
		University postgraduate	22		

**Number of parents in paid work**	7906	0	17		
		1	41		
		2	42		

**Parents' ethnic background**	6352	Norwegian	89		
		Western or mixed	8		
		Non-Western	2		

**Native language**	7718	Norwegian	95		
		Sami	0		
		Other Nordic	1		
		Other European	2		
		Non-European	2		

^**a**^World Health Organization [20,21]

Table 3 gives the distribution of the variables related to the clinics' accessibility and involvement of the parents. These include length of treatment period and the recorded waiting time, taken from the clinics' registers, and the following six items from the questionnaire: 1) Perceived waiting time, 2) number of consultations in the previous 3 months, 3) having received a suitable number of consultations in the previous 3 months, 4) ease of contacting the therapist outside appointments, 5) parents' participation in consultations and 6) parents' understanding of the services.

**Table 3:** Descriptive statistics for the clinics' accessibility and involvement of the parents

	*n*		%	Mean (SD)	Median
Length of treatment (days)	7903			436.5 (531.7)	256

Recorded waiting time (days)	7902			83.1 (82.7)	55

Perceived waiting time	7700	None	13		
		Not for long	44		
		Fairly long	22		
		Very long	21		

Number of consultations in the previous 3 months?	7173	Just one	35		
		2-5	46		
		6-12	17		
		More than 12	2		

Suitable number of consultations in the previous 3 months	7361	No, far too few	14		
		No, too few	21		
		Yes, a suitable number	65		

Ease of contacting the therapists outside appointments	5966	Not at all	9		
		To a small extent	16		
		To a moderate extent	35		
		To a large extent	29		
		To a very large extent	11		

Parents' participation in consultations	7580	No, never	13		
		Yes, occasionally	49		
		Yes, often	39		

Parents' understanding of the services	7547	Very poor	4		
		Quite poor	7		
		Neither poor nor good	21		
		Quite good	42		
		Very good	26		

The results of the multiple regression analyses are presented in table 4. The adjusted *R*^2 ^values for the full model were 0.43 for the "Relationship with health personnel" scale, 0.41 for the "Information and participation" scale and 0.20 for the "Outcome" scale. Altogether the demographic and clinical variables explained 2-4% of the total variance of each of the three scales. Thus, the organizational variables accounted for most of the explained variance.

**Table 4:** Regression results: β coefficient^*a*^, *p*^*b *^and cumulative adjusted *R*^2 ^(Adj *R*^2^) for each block entered

	Relationship with health personnel			Information and participation			Outcome		
**Independent variables**	**β**	***p***	**Adj R**^**2**^	**β**	***p***	**Adj R**^**2**^	**β**	***p***	**Adj R**^**2**^

(Intercept)	*46.76*	*0.000*		*27.36*	*0.000*		*78.02*	*0.000*	

**CHILD'S DEMOGRAPHIC DATA**			**0.004**			**0.010**			

**Child's gender **(ref Female)									
Male				0.050	0.001				

**Child's age**	-0.063	0.000		-0.014					

**CHILD'S CLINICAL DATA AND PREVIOUS CONTACT WITH CAMHS**			**0.009**			**0.021**			**0.034**

**Diagnosis axes 1,2,3 and 5 **(ref hyperkinetic or conduct disorders)									
Emotional disorders	-0.029			-0.106	0.000		-0.033		
Developmental disorders	0.015			-0.014			-0.103	0.000	
Psychosocial problems only	0.013			-0.043	0.005		-0.080	0.000	
No mental or behavioural disorders or psychosocial problems	0.009			-0.023			-0.077	0.000	
Other mental or behavioural disorders	0.008			-0.021			-0.059	0.001	
Not coded or invalid code used	-0.018			-0.023			-0.044	0.013	

**Axis 6 **(Global Assessment of Psychosocial Disability scale)	-0.029			0.007			-0.030		

**Previously treated by CAMHS**	-0.030						-0.074	0.000	

**PARENTS' DEMOGRAPHIC DATA**			**0.019**			**0.021**			**0.036**

**Parents' gender **(ref Female)									
Male	-0.005			0.007					

**Parents' age**	0.047	0.006		-0.017					

**Marital status **(ref Married)									
Cohabiting	-0.010			-0.018					
Neither married nor cohabiting	-0.040	0.035		-0.032					

**Household's highest education**				-0.052	0.000		-0.087	0.000	

**Number of parents in paid work**	-0.015			-0.015			0.021		

**Parents' ethnic background **(ref Norwegian)									
Western or mixed	0.038	0.012					0.009		
Non-Western	0.002						0.022		

**Native language **(ref Norwegian)									
Other Nordic				0.018					
Other European				0.032	0.020				
Non-European				0.021					

**ACCESSIBILITY AND INVOLVEMENT OF THE PARENTS**			**0.425**			**0.407**			**0.199**

**Length of treatment**	-0.034	0.044		0.026			0.062	0.001	

**Recorded waiting time**							-0.022		

**Perceived waiting time**	-0.073	0.000		-0.043	0.004		-0.026		

**Number of consultations in the previous 3 months**	0.059	0.000		-0.031	0.040		-0.132	0.000	

**Suitable number of consultations in the previous 3 months**	0.191	0.000		0.154	0.000		0.186	0.000	

**Ease of contacting the therapist outside appointments**	0.263	0.000		0.193	0.000		0.086	0.000	

**Parents' participation in consultations**	0.040	0.013		0.027			-0.036		

**Parents' understanding of services**	0.326	0.000		0.429	0.000		0.248	0.000	

^a ^Blank spaces in the β-coefficient column indicate that the variable was not included in the final model because it had no statistically significant effect (*p *> 0.1) in the bivariate regression analysis.

^b ^Blank spaces in the *p *column indicate that the variable was not statistically significant (*p *> 0.05).

The child's age was significantly negatively correlated with the "Relationship with health personnel" scale. On the "Information and participation" scale, parents of boys were significantly more satisfied than parents of girls. However, these variables explained only a small amount of the variation. Both of these variables were left out of the multivariate analysis for the "Outcome" scale because none of them were significantly related with this scale in the bivariate linear regression analysis.

None of the clinical variables significantly affected the "Relationship with health personnel" scale. For the other two scales, parents of children with hyperkinetic or conduct disorders tended to be more positive than the others. Parents of children previously treated by CAMHS were statistically significantly more negative on the "Outcome" scale. The clinical data explained slightly more than 3% of the explained variation on "Outcome" scale, and had even less explanatory power on the other two scales.

The parents' background explained only a small amount of the variation on the "Relationship with health personnel" scale. Older and married parents and parents with a Western or mixed background were more positive. These variables did not add to the explained variance on the "Information and participation" scale and only marginally to that on the "Outcome" scale, even though higher education predicted less satisfaction on both, and non-Nordic Europeans were significantly more positive than Norwegians on the "Information and participation" scale.

The organizational variables accounted for most of the variation explained by our models, increasing the adjusted *R*^2 ^values from 2% to 43% for the "Relationship with health personnel" scale, from 2% to 41% for the "Information and participation" scale and from 4% to 20% for the "Outcome" scale. Most of these independent variables exerted statistically significant effects on all three scales. The exceptions were the following four variables: 1) "Length of treatment" had no statistically significant effect on the "Information and participation" scale, 2) "Recorded waiting time" was included only on the "Outcome" scale and did not show a statistically significant effect on this scale, 3) "Perceived waiting time" had no statistically significant effect on the "Outcome" scale, and 4) "Parents participate in consultations" had no statistically significant effect on the "Information and participation" or *"*Outcome" scale.

On all scales, three variables had considerably higher β values than the others, two of which were common to all three scales. Firstly, the variable "Parents' understanding of the services" had the highest β value on all the three scales: 0.326, 0.429 and 0.248 for "Relationship with health personnel", "Information and participation" and "Outcome", respectively. Secondly, parents who answered that they had a "Suitable number of consultations" in the previous 3 months were more positive than parents who answered that they had less than a suitable number of consultations. The β values were 0.191, 0.154 and 0.186 for "Relationship with health personnel", "Information and participation" and "Outcome", respectively. For the "Relationship with health personnel" and "Information and participation" scales, "Ease of contacting the therapist outside appointments" had the second highest β values (0.263 and 0.193, respectively), indicating a higher score as a result of easier access. "Number of consultations in the previous 3 months" had the third highest β value on the "Outcome" scale (-0.132), predicting that a lower number of consultations is associated with a better perceived outcome.

## Discussion

In this study we evaluated the predictors of parents' experiences with three aspects of outpatient CAMHS. Organizational aspects of the clinic, such as the parents' perceived accessibility and involvement, were much stronger predictors than were demographic and clinical variables. This is in accordance with another study that found that parental satisfaction is predicted by the degree to which clinics are able to meet the parents' desires and expectations [22].

"Parents' understanding of the services" had the highest β value on the three scales. The "Information and participation" scale addressed the parents' experience with the information about the child's condition, treatment and the parents' influence on the choice of treatment. It seems reasonable that a high score on these variables implies that parents feel they have a good understanding of the services. The "Relationship with health personnel" scale included a question about the cooperation between health personnel and parents, and whether health personnel speak in a way that is understandable. A high score on these questions is likely to lead to a high score on the understanding of the services. The parents understanding of the services also predicted a high score on the "Outcome" scale, which is about changes in the child's condition and social functioning. The time it takes to gain a good understanding of the services might also be associated with the time needed to see a positive outcome. A good understanding of the services might also imply a more realistic expectation about the degree and speed of improvement.

It can be argued that the "Parents' understanding of the services" itself is an outcome of the "Relationship with health personnel" and "Information and participation" scales, and that it should not be included as an independent variable. This does not apply to the "Outcome" scale. Initial analyses with this independent variable left out did not change the overall results, and the major part of the explained variance was still attributable to the organizational data.

Having experienced a "Suitable number of consultations in the previous 3 months" was a stronger predictor on all three scales than the actual number of consultations. It seems intuitively correct that a less-than-suitable number of consultations in the previous 3 months would predict a lower score on all three scales. On the other hand, a higher number of consultations in the previous 3 months also predicted lower scores on the "Outcome" and "Information and participation" scales. This apparently contradictory finding may be explained by considering the severity of the child's problem. It seems reasonable that a more severe condition elicits a greater effort from the clinic. It is also known from studies of patient satisfaction in general that a poorer health status is associated with a lower satisfaction score [10,15]. Our data also suggest that the frequency of consultations is highest at the onset of treatment. Parents in the early stage of treatment may not yet have reached the point where a notable outcome may be observed, or they may have not yet received sufficient information. It has also been suggested that dissatisfied patients drop out of treatment more quickly than others [23], and will thus not be among the long-term users with a lower frequency of consultations. Clearly this is a complex question that needs further investigation.

Neither recorded nor perceived waiting time significantly affected the "Outcome" scale, but a longer perceived waiting time predicted lower scores on the other two scales. This suggests that parents who have experienced a long waiting time develop a negative attitude that is difficult to change.

The "Outcome" scale addresses changes in the child's condition and social functioning, while the other two scales have more of a relational and communicative character. This makes it more plausible that the child's diagnosis, "Previous contact with CAMHS" and "Length of treatment" have a stronger effect on the "Outcome" scale than on the other two scales. The observation that previous contact with CAMHS predicts a lower score on the "Outcome" scale may indicate that the child's condition is severe or chronic, with little perceived improvement.

### Limitations of the study

This study considered only the experiences of the parents, and not those of the children. However, there is evidence that the parents' perception of the services is correlated to their children's perception only to a limited degree [2,14], and that children are more negative than their parents [2,16]. Other studies have shown that the correlation between parents' and child's satisfaction increases with the age of the child [24,25]. However, the parents' perspective represents more than a proxy for the children's experiences, since parents often form an integral part of the treatment process.

Clinical data beyond the main diagnosis for each of the six axes were not available for this study. Information about comorbidity and multi-informant assessment of the child's condition could have increased the apparent importance of the child's condition as a predictor of parental experiences. Assessments before and after treatment might have provided a more valid measure of treatment outcome.

The results may have been influenced by non-Norwegian respondents being underrepresented in the sample; although they represent a small group at the national level, they may constitute a relatively large proportion of the patients in certain areas. The questionnaire was distributed only in Norwegian, and so parents experiencing the greatest linguistic barriers in living in Norway were unable to respond to this survey. Nevertheless, to the extent that the ethnic background or native language had an effect, Norwegians were less satisfied. But again, this explains only a minor part of the variation in this material, and there is little support for ethnic background as a predictor for parental satisfaction with outpatient CAMHS in the literature.

### Strengths of the study

One strength of this study is its comprehensiveness. All 86 outpatient CAMHS units in Norway participated, with a total number of 7906 respondents. Various background variables were collected from the clinics' registers and the parents' self-reports. The parents' evaluations of the services were collected with a questionnaire with satisfactory psychometric properties [19].

The response rates in patient experience surveys in CAMHS have generally been low [3], but it has been shown that reminders are effective at increasing response rates [15]; in this study the non-respondents received two reminders. A response rate of 46% may seem low, but the follow-up study of the non-responders revealed that the results are representative of the entire population.

## Conclusions

The parents' assessment of their experiences with CAMHS is strongly predicted by variables related to the clinics' accessibility and the involvement of the parents. The demographic and clinical characteristics have less explanatory power. The most important single item for all three scales is the parents' understanding of the services. In addition, the perception of being offered a suitable number of consultations and having easy access to the therapist outside appointments has a strong predictive effect on the parents' experiences on the three aspects of the services assessed in this study. These variables are at least partly controlled by the clinics themselves, and clinics could therefore enhance parental satisfaction by reducing the waiting time, being accessible during treatment, involving the parents and being attentive to their concerns.

## Competing interests

The authors declare that they have no competing interests.

## Authors' contributions

OH led the development of the questionnaire and was involved in data acquisition. All authors contributed to the design of the study. OH conducted the analyses and drafted the manuscript. All authors have made significant contributions by critically reviewing the manuscript and have read and approved the final version.
